# What Shall We Do about Porcelain?

**Published:** 1904-03

**Authors:** 


### WHAT SHALL WE DO ABOUT PORCELAIN?

---

If I may be permitted at this point, I will relate my own experience in this respect. I will say that I had a Hammond furnace in my office for fully a year before I made my first inlay, waiting patiently for the time when I had leisure moments that might be improved by taking the first steps in porcelain work.

I made my first inlay a few months ago out of what seemed to be sheer necessity. A young patient presented with the mesial third of a central incisor broken off. Three gold fillings had failed successively all within a few months, each time breaking an additional portion of tooth structure. The adjoining teeth were sound and beautiful, and the disfigurement that must follow the insertion of a gold filling so impressed me that I asked her to wait a week or so, in the meantime I would decide how best to restore the broken corner.

I canceled my work for an entire afternoon and went to work. I completed the inlay and attempted to set it, but used too much pressure laterally and scaled off a layer of porcelain. Two days later I made another to replace the broken one, in the meantime modifying the shape of my cavity, providing a more definitely-outlined seat for the inlay.

Since that time or during the past five months I have inserted something over eighty inlays. Of these three have come out, due to faulty cavity preparation. Two or three others I am going to replace. From the majority of the balance I look for a fair degree of successful service. I keep a special record of my inlays and will examine them by appointment at stated times and record the condition I find each time. In this way I hope to keep close watch of my failures and perhaps learn by hard experience a few of the many things regarding porcelain work that many of the experts have been attempting to teach us through the dental journals during the past two or three years.

So far, I have said nothing about the two factions, so to speak, namely the advocates of high or low-fusing bodies.

Regarding the low-fusing body or the gold matrix, Dr. Jenkins, of Dresden, probably its greatest exponent, in a paper read before one of the European dental societies, makes the statement that he believes that no one within the sound of his voice would think of using a material so intractable as platinum for obtaining a matrix.

To this Dr. Reeves replies in a paper read before the Iowa State Dental Society, 1902, referring to the Jenkins system, he says: "The one great point the Jenkins followers make, about the only one they have a shadow of a chance to make, is the greater ease with which gold can be burnished into a cavity to form a matrix, but the greater ease and safety with which a platinum matrix can be handled afterward more than counterbalances both in time saved and in accuracy of results obtained. I know of no cavity in which gold could be burnished that platinum could not be burnished better, and I can call to mind several in which, if gold were used, it would have been so distorted in removal from the cavity as to be useless as a matrix."

Further on Dr. Reeves says, "I will burnish a matrix from start to finish in from fifteen to twenty minutes."

After the matrix is obtained there would seem to be little opportunity for argument regarding what body should best be used. The principles of building up an inlay in layers as first worked out and demonstrated by Dr. Reeves, seems so much in advance in its results of anything I have seen in similar cases, by use of the low-fusing body, that I would consider it a practical waste of time at least to give any special attention to the low-fusing porcelains.

But to return for a moment to the principal point I am especially anxious to emphasize at this time, I wish to say that the busy dentist should use porcelain whenever it is indicated and devote as many sittings to each operation as the conditions demand. Make yourself master of every situation and the appreciation from your patients that inevitably follows will more than repay for the efforts made.

Remember this, your patients will have confidence in your efforts in direct proportion as you show interest in their welfare. When you have developed that degree of skill in the manipulation of this material to which you aspire, your fees will be in proportion to the skill displayed and the results obtained.—*Register.*

